# Early Endothelial Injury in Pancreas Transplantation: Insights from a Prospective Cohort Largely Composed of Simultaneous Pancreas-Kidney Recipients

**DOI:** 10.3390/medsci14020241

**Published:** 2026-05-06

**Authors:** Joana Ferrer-Fàbrega, Andrea Llaves-López, Ramón Rull, Ángeles García-Criado, Pedro Ventura-Aguiar, Rocío García-Pérez, Martí Manyalich-Blasi, Antonio J. Amor, José Ríos, Fritz Diekmann, Josep Fuster, Emma Folch-Puy

**Affiliations:** 1 Hepatobiliopancreatic Surgery and Liver and Pancreatic Transplantation Unit, Department of Surgery, Institute Clínic of Digestive and Metabolic Diseases (ICMDiM), Hospital Clínic, Universitat de Barcelona, 08036 Barcelona, Spain; joferrer@clinic.cat (J.F.-F.); rrull@clinic.cat (R.R.); rgarcia5@clinic.cat (R.G.-P.); jfuster@clinic.cat (J.F.); 2 Network for Biomedical Research in Hepatic and Digestive Diseases (CIBEREHD), 08036 Barcelona, Spain; magarcia@clinic.cat (Á.G.-C.); pventura@clinic.cat (P.V.-A.); fdiekman@clinic.cat (F.D.); 3 Institut d’Investigacions Biomèdiques August Pi i Sunyer (IDIBAPS), 08036 Barcelona, Spain; ajamor@clinic.cat (A.J.A.); jose.rios@uab.cat (J.R.); 4 Department of Experimental Pathology, Instituto de Investigaciones Biomédicas de Barcelona (IIBB), Consejo Superior de Investigaciones Científicas CSIC, 08036 Barcelona, Spain; andrea.llaves@iibb.csic.es; 5 Doctoral Program in Biomedicine, Universitat de Barcelona, 08036 Barcelona, Spain; 6 Department of Radiology, Hospital Clínic, Universitat de Barcelona, 08036 Barcelona, Spain; 7 Renal Transplant Unit, Nephrology and Kidney Transplant Department, Hospital Clínic, Universitat de Barcelona, 08036 Barcelona, Spain; 8 Unit of Medical and Surgical Endocrinology, Endocrine Surgery, Department of General and Digestive Surgery, Hospital Clinic Barcelona, Universitat de Barcelona, 08036 Barcelona, Spain; manyalich@clinic.cat; 9 Diabetes Unit, Department of Endocrinology and Nutrition, Hospital Clínic, Universitat de Barcelona, 08036 Barcelona, Spain; 10 Department of Clinical Pharmacology, Hospital Clinic and Medical Statistics Core Facility, Universitat de Barcelona, 08036 Barcelona, Spain; 11 Biostatistics Unit, School of Medicine, Universitat Autònoma de Barcelona, 08193 Barcelona, Spain

**Keywords:** simultaneous pancreas-kidney transplantation, pancreas retransplantation, diabetes mellitus, ischemia–reperfusion injury, endothelial dysfunction, glycocalyx, graft survival, post-transplant complications

## Abstract

Background/Objectives: Ischemia–reperfusion injury (IRI) contributes to graft dysfunction in solid organ transplantation, with the pancreas vulnerable due to its fragile vasculature. Endothelial glycocalyx (eGCX) disruption is central to this process. This study prospectively examined perioperative endothelial injury in pancreas transplantation. Methods: Fifty-two recipients were included, of whom 47 underwent simultaneous pancreas-kidney (SPK) transplantation and 5 pancreas retransplantation. Biomarkers of eGCX degradation (syndecan-1, heparan sulfate (HS) and hyaluronan) and endothelial injury (soluble thrombomodulin, VEGF and soluble VEGFR1) were measured in plasma preoperatively, 10 min after pancreas reperfusion, 24 h later, and at discharge. Associations with donor type and early post-transplant outcomes were explored. Results: A marker endothelial injury was evident within 10 min of pancreas reperfusion, before kidney implantation, characterized by increased syndecan-1, HS, and sVEGFR1, together with decreased VEGF. Hyaluronan peaked at 24 h, consistent with a broader systemic endothelial response. Controlled donation after circulatory death donors showed higher syndecan-1 levels at 10 min PR and higher VEGF at 24 h. Seven recipients developed pancreas graft loss, which was linked to lower VEGF at 10 min post-reperfusion and lower hyaluronan levels both before surgery and at discharge. Kidney acute tubular necrosis was related with higher preoperative HS and elevated 24 h sVEGFR1. Among recipients with functioning grafts, preoperative endothelial biomarkers were linked to postoperative complications. Conclusions: Pancreas transplantation triggers early endothelial injury and glycocalyx shedding, particularly in a predominant SPK setting. Perioperative endothelial biomarkers may have a value for early risk stratification after transplantation.

## 1. Introduction

Diabetes caused over two million deaths in 2021, according to a global report by the World Health Organization [1]. To date, pancreas transplantation remains the most effective therapy for type 1 diabetes mellitus (DM), improving survival and quality of life [2]. However, the number of procedures has declined due to donor shortage, [3] and ischemia–reperfusion injury (IRI) continues to compromise graft function and survival [4].

The pancreas has a complex vascular architecture and functions as a low-flow organ in vivo [5]. Therefore, high perfusion pressures can induce endothelial damage and edema, whereas insufficient pressure may lead to inadequate perfusate distribution and suboptimal perfusion. These characteristics render pancreatic grafts particularly susceptible to IRI during transplantation, increasing the risk of complications and graft loss compared to other solid organ transplants [6].

In simultaneous pancreas-kidney (SPK) transplantation, the ischemic insult also involves the renal graft. In kidney transplantation, IRI is a major determinant of delayed graft function (DGF) and acute tubular necrosis (ATN), and endothelial and microvascular damage are recognized as central mechanisms in this process. [7,8]. Thus, in SPK recipients, both grafts are exposed to peri-transplant ischemic and inflammatory burden, in which endothelial dysfunction likely contributes to early post-transplant outcomes.

Endothelial injury represents a shared pathogenic mechanism across solid organ transplantation [9]. The luminal surface of the endothelium is coated with a dynamic layer known as the endothelial glycocalyx (eGCX), which forms the primary interface between circulating blood and the endothelium. eGCX is composed of membrane-bound proteoglycans, associated glycosaminoglycans, and glycoproteins that help preserve endothelial integrity [10], but deteriorate under conditions such as DM, IRI, and sepsis, triggering the shedding and release of its components into the bloodstream and urine [11]. eGCX breakdown products have been detected after liver [12], kidney [13], heart [14], and lung [15] transplantation.

eGCX also binds and incorporates plasma-derived molecules, including albumin, antioxidant enzymes, growth factors, and membrane-anchored proteins such as thrombomodulin (TM), vascular endothelial growth factor (VEGF), and its receptor, VEGF receptor 1 (VEGFR1). Together, the eGCX and plasma constituents comprise the endothelial surface layer, which plays a crucial role in maintaining homeostasis [11].

Despite the central role of endothelial preservation in graft survival, little is known about its contribution to pancreatic IRI. This study aimed to evaluate endothelial integrity and function during transplantation and to investigate their association with post-transplant complications.

## 2. Materials and Methods

### 2.1. Study Design

This prospective observational study included 52 consecutive patients undergoing pancreas transplantation at the Hospital Clínic de Barcelona between March 2021 and August 2023: 47 SPK transplants and 5 pancreas retransplantations. Sixteen healthy volunteers served as controls. The study was approved by the Institutional Review Board (HCB/2020/0499, date of approval: 28 May 2020) and conducted in accordance with the Helsinki Declaration of 1975 [16].

### 2.2. Donor Characteristics

Pancreas graft acceptance followed National Transplant Organization criteria (2005, updated 2018) [17]. Donor variables included age, gender, type of donor, cause of death, body mass index, pancreas and kidney cold ischemia time, preservation solution and days at intensive care unit.

During organ procurement, both the abdominal aorta and the inferior mesenteric vein were cannulated for organ perfusion with standard preservation solutions: University of Wisconsin, Institut Georges Lopez-1, Celsior and Histidine-Tryptophan-Ketoglutarate. The standard whole-pancreas graft included the entire pancreas and the duodenal segment.

### 2.3. Recipient Characteristics

All recipients had type 1 DM and met the inclusion criteria defined by the protocol established in Hospital Clínic de Barcelona [18]. Demographic and clinicopathological features evaluated included age, gender, body mass index, duration of diabetes (DM vintage), duration of dialysis (dialysis vintage), type of dialysis, type of transplant, method of arterial anastomosis, hospital stay and laboratory parameters.

### 2.4. Surgical Procedure

The pancreas was harvested en bloc along with the donor duodenum and spleen. The graft was subsequently prepared on the back table and placed intraperitoneally on the right side of the pelvis. Venous systemic drainage was performed between the graft portal vein and recipient vena cava. Arterial supply for the pancreatic graft was established through the anastomosis of the recipient right iliac primitive artery to the graft superior mesenteric artery or the common iliac graft artery, depending on the back table reconstruction [19]. For exocrine secretion, enteric drainage was created “side-to-side”, by duodeno-duodenostomy anastomosis.

In addition, surgical complications were defined according to the modified Clavien-Dindo classification as any postoperative event related to the procedure within 90 days after the transplant [20]. Postoperative hemorrhage was classified according to the definition of the International Study Group for Pancreatic Surgery (ISGPS) [21]. Due to the lack of a clear consensus on the definition of graft pancreatitis, cases were considered as such when it was evident that the condition had arisen intraoperatively from IRI and its related complications, including pancreatic abscesses and peripancreatic fluid collections. Other conditions were also considered, such as sterile or infected abdominal fluid collection, identified either through ultrasound/abdominal computed tomography or based on clinical symptoms. Intestinal complications included duodenum-related leaks and small-bowel obstruction.

Kidney DGF was defined as the need for at least one session of hemodialysis during the first week following transplantation. Kidney ATN is an ischemic or nephrotoxic tubular injury causing delayed graft function in the absence of acute rejection. Postoperative kidney surgical complications were defined as complications occurring within the first 3 months after transplantation, excluding DGF and ATN. Early pancreatic graft function was evaluated using biochemical parameters (serum amylase and lipase levels together with insulin requirements) and clinical outcomes, including the need for transplantectomy within 30 days of transplantation.

### 2.5. Immunosuppression

Immunosuppressive therapy was administered in accordance with institutional protocols, which varied according to the transplantation date. Standard induction therapy consisted of either an anti-interleukin-2 receptor monoclonal antibody (basiliximab) or a rabbit anti-human thymocyte polyclonal antibody (thymoglobulin). Subsequent maintenance immunosuppression was based on triple therapy with calcineurin inhibitor (tacrolimus), mycophenolate, and steroids (methylprednisolone in the immediate post-transplant period, followed by oral prednisone) [22].

### 2.6. Anticoagulant Therapy and Antibiotic Prophylaxis

The standard anticoagulation protocol consisted of enoxaparin 20 mg every 12 h, starting 8 h after surgery and continuing until hospital discharge, provided that there were no thrombotic or hemorrhagic complications. Acetylsalicylic acid was initiated 12 h post-surgery at a dose of 50 mg/day until discharge, when it was increased up to 100 mg/day. The perioperative antibiotic prophylaxis included vancomycin and ertapenem. Universal antifungal prophylaxis with fluconazole was administered to all the recipients. Cytomegalovirus prophylaxis was provided with either ganciclovir or valganciclovir, depending on the patient’s glomerular filtration rate.

### 2.7. Data Collection and Blood Sampling

Demographic and clinicopathological data were obtained from patients’ medical records. Blood samples were collected at four different time points: before surgery (BS), to determine baseline levels of diabetic patients; at 10 min post-reperfusion (PR), a critical time point known to reflect peak IRI; at 24 h PR of the graft, to capture early but sustained effects of IRI; and at hospital discharge. Patients were discharged once their creatinine levels indicated adequate kidney function, thereby minimizing the potential bias in the analysis. Blood was drawn into vacuum tubes (BD Vacutainer PPT, BD, Franklin Lakes, NJ, USA) and centrifuged at 1100× *g* for 11 min (Hettich EBA 270, Andreas Hettich GmbH & Co. KG, Tuttlingen, Germany). Plasma was subsequently collected and stored at −80 °C until analysis.

In SPK transplantation, the pancreas graft was implanted and reperfused before kidney implantation. Accordingly, the 10 min PR blood sample was obtained after pancreas reperfusion and before renal graft implantation, whereas the 24 h PR sample reflects the post-transplant systemic response after both grafts had been implanted. Because most pancreas transplants in our cohort were performed as SPK, interpretation of circulating endothelial biomarkers required consideration of the shared but not identical ischemia–reperfusion burden affecting both grafts. Importantly, this sequential reperfusion pattern means that later systemic biomarker changes cannot be assumed to represent the additive contribution of two independently reperfused organs. This temporal sequence is relevant for interpretation of perioperative biomarker dynamics.

### 2.8. Assessment of Endothelial Integrity and Injury Markers

Plasma concentrations of eGCX degradation products syndecan-1, heparan sulfate (HS), and hyaluronan, as well as endothelial injury markers, including soluble TM (sTM), VEGF, and soluble VEGFR1 (sVEGR1), were quantified using enzyme-linked immunosorbent assay (ELISA) kits, following the manufacturer’s instructions. The kits used were syndecan-1 (Diaclone, Besançon, France; 950.640.192), HS (Elabscience, Houston, TX, USA; E-EL-H2364) and hyaluronan, VEGF, sTM, and sVEGFR1 (R&D Systems, Abingdon, UK; DHYAL0, DVE00, DTHBD0, and DVR100C, respectively). All measurements were performed in duplicates.

### 2.9. Statistical Analysis

Given the exploratory and descriptive nature of this study, formal power calculation was not applicable and multivariate analyses could not be performed due to the low number of pancreatic graft loss events. The sample size was determined based on historical data from the Spanish National Transplant Organization to ensure comprehensive representation of the target population. Between 2018 and 2021, approximately 21 pancreas transplants were performed annually at the Hospital Clínic Barcelona [23]. Statistical analyses were performed using GraphPad Prism version 10 (GraphPad Software Inc., San Diego, CA, USA) and SPSS Statistics version 29 (SPSS Inc.; Chicago, IL, USA). Categorical variables were expressed as frequencies (percentages) and continuous variables as medians with interquartile ranges (25th–75th percentile) [IQR]. Between-group comparisons were performed using appropriate non-parametric tests for continuous variables and chi-square (χ^2^) or Fisher’s exact test for categorical variables, as appropriate. The evolution of the endothelial biomarkers syndecan-1, HS, hyaluronan, sTM, VEGF, and sVEGFR1 was evaluated over time to assess differences between donor types: donor after brain death (DBD) and donor after circulatory death (cDCD). Transplant outcomes such as pancreatic graft loss and acute tubular necrosis (ATN) were analyzed using generalized estimating equation (GEE) models to account for intra-subject correlation, employing an autoregressive AR(1) correlation structure. Owing to the distributional characteristics of the studied biomarkers, these analyses were performed using a non-parametric approach by means of rank transformation. *p*-values of less than 0.05 were considered statistically significant.

## 3. Results

### 3.1. Demographic and Clinicopathological Characteristics of Donors and Recipients

Donor and recipient characteristics are shown in Table 1 and Table 2. Donors were predominantly young males. The majority of donors were classified as DBD, with trauma and cerebrovascular accidents as leading causes of death. Institut Georges Lopez-1 was the most frequently used preservation solution. Median cold ischemia times were within accepted ranges for both pancreas and kidney grafts. Recipients were predominantly male patients with long-standing type 1 DM, most of whom were on dialysis at transplantation. Postoperative biochemical markers reflected tissue injury patterns at 24 h PR.

### 3.2. Circulating Tissue Injury Markers

**Figure 1:**
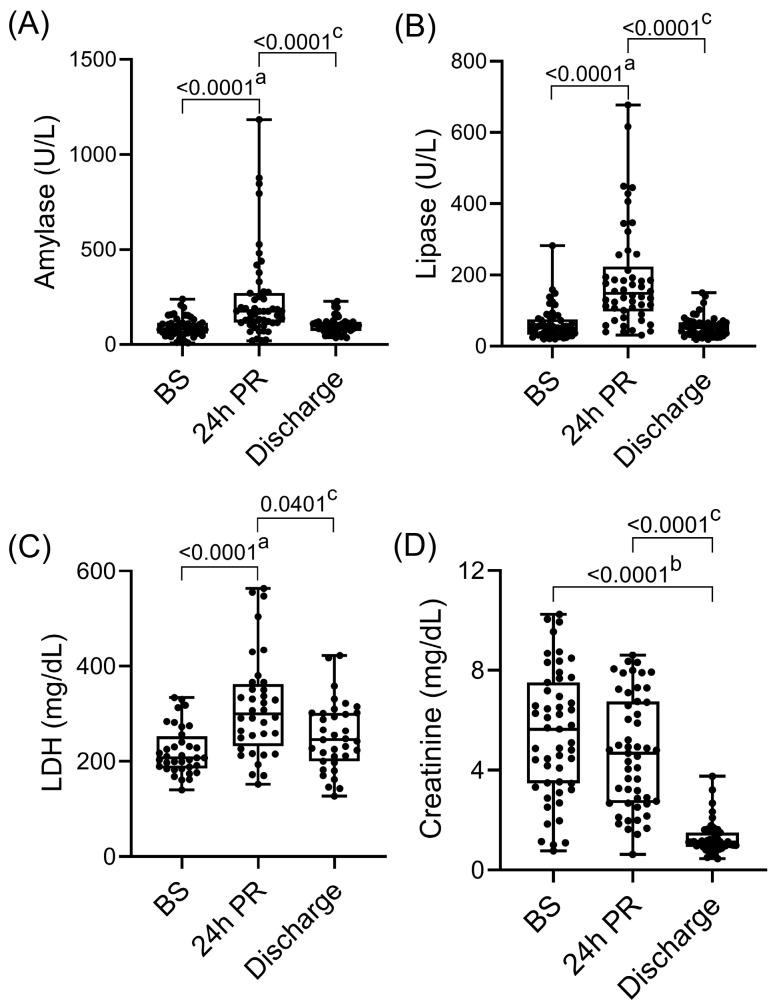
Plasma levels of tissue injury markers. (**A**) amylase; (**B**) lipase; (**C**) LDH and (**D**) creatinine. Boxes represent the interquartile ranges and whiskers extend to the minimum and maximum values. The median values are shown within the boxes. Each dot represents one individual patient. *p*-values of less than 0.05 were considered statistically significant. ^a^ BS vs. 24 h PR, ^b^ BS vs. Discharge, ^c^ 24 h PR vs. Discharge. PR indicates post-reperfusion; LDH, lactate dehydrogenase.

As shown in Figure 1, amylase and lipase significantly increased at 24 h after reperfusion (amylase: 82 [57.3–115.3] U/L vs. 175 [116.5–271.3] U/L; *p* < 0.0001; lipase: 51.5 [31.5–76.5] U/L vs. 148.5 [98.3–223.8] U/L; *p* < 0.0001) and declined to near-normal levels by discharge (amylase: 175 [116.5–271.3] U/L vs. 97.5 [72.8–120] U/L; *p* < 0.0001; lipase: 148.5 [98.3–223.8] U/L vs. 52.5 [33–67.5] U/L; *p* < 0.0001). Similarly, the levels of non-specific cell injury marker LDH showed a transient postoperative increase (207 [185–252] mg/dL vs. 300 [231.8–362.3] mg/dL, *p* < 0.0001) and approached baseline at discharge (300 [231.8; 362.3] mg/mL vs. 245 [200; 302] mg/dL; *p* = 0.0401). Creatinine levels progressively decreased (5.6 [3.5, 7.5] mg/dL BS vs. 1.1 [0.9, 1.5] mg/dL at discharge; *p* < 0.0001), consistent with recovery of kidney graft function.

### 3.3. Circulating Endothelial Injury Markers

**Figure 2:**
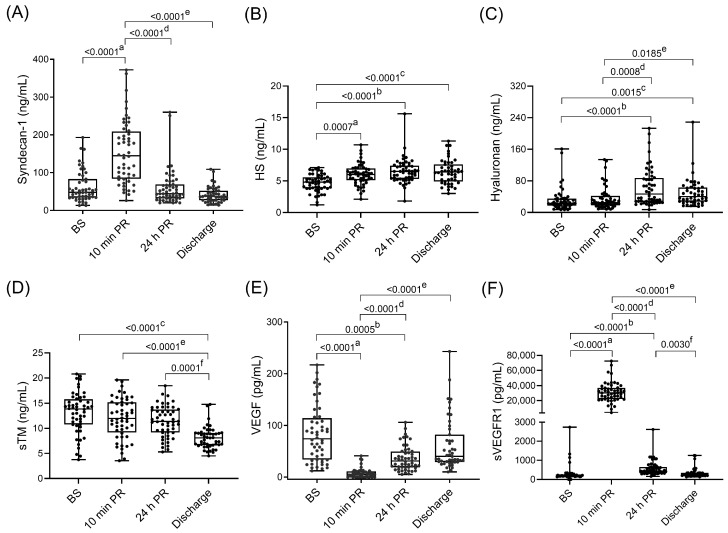
Plasma levels of endothelial injury markers over time. (**A**) Syndecan-1; (**B**) HS; (**C**) Hyaluronan; (**D**) sTM; (**E**) VEGF and (**F**) sVEGFR1. Boxes represent the interquartile ranges and whiskers extend to the minimum and maximum values. The median values are shown within the boxes. Each dot represents one individual patient. *p*-values of less than 0.05 were considered statistically significant. ^a^ BS vs. 10 min PR, ^b^ BS vs. 24 h PR, ^c^ BS vs. Discharge, ^d^ 10 min PR vs. 24 h PR, ^e^ 10 min PR vs. Discharge, and ^f^ 24 h PR vs. Discharge. No *p*-value is shown when ≥0.05. BS indicates before surgery; PR, post-reperfusion; HS, heparan sulfate; sTM, soluble thrombomodulin; VEGF, vascular endothelial growth factor; sVEGFR1, soluble vascular endothelial growth factor receptor 1.

Plasma levels of the three major components of the eGCX significantly increased within the first 24 h of PR compared to baseline values (Figure 2A–C): syndecan-1 (48 [34; 83] vs. 145 [85; 207] ng/mL; *p* < 0.0001) and HS (4.8 [3.8; 5.6] vs. 6 [5.1; 7] ng/mL; *p* = 0.0007) levels rose rapidly at 10 min PR, whereas hyaluronan peaked at 24 h (23 [17; 35] vs. 46.5 [26.5; 87.2] ng/mL; *p* < 0.0001).

Among endothelial injury markers (Figure 2D–F), sVEGFR1 showed a marked transient increase at 10 min PR (202 [172; 264] vs. 30,130 [21,928; 37,088] pg/mL; *p* < 0.0001), while VEGF levels decreased at the same time point (74 [34; 114] vs. 5 [0; 11] pg/mL; *p* < 0.0001). sTM decreased progressively throughout the transplantation process, from 13.9 [10.8; 15.8] ng/mL at BS to 8.1 [6.6; 9.1] ng/mL at discharge; *p* < 0.0001.

Compared with healthy controls, diabetic patients (Table 3) exhibited higher baseline levels of syndecan-1 (29.5 [24.7; 40] vs. 48 [34; 83] ng/mL; *p* = 0.013), sTM (3.2 [2.6; 3.5] vs. 13.9 [10.8; 15.8] ng/mL; *p* < 0.0001), and VEGF (23 [17.7; 48.7] vs. 74 [34; 114] pg/mL; *p* = 0.015). The percentage increases for syndecan-1 and VEGF were 69.8 and 72%, respectively. No significant differences were observed for hyaluronan or sVEGFR1. In contrast, HS levels were significantly higher in healthy controls than in transplant recipients at baseline (6.3 [5.3, 7.1] vs. 4.8 [3.8, 5.56] ng/mL; *p* = 0.003).

### 3.4. Endothelial Injury Markers According to Donor Type, Pancreas Graft Loss, and Kidney Acute Tubular Necrosis

Recipients of grafts from cDCD donors showed significantly higher PR plasma levels of syndecan-1 and VEGF compared with DBD donors (Table 4). Lower VEGF levels at 10 min PR and reduced baseline and discharge hyaluronan levels were significantly associated with early pancreas graft loss. Out of the seven individuals experiencing pancreatic graft failure, five presented early loss within the first postoperative month, each requiring transplantectomy. Of the remaining two, one patient developed a pancreatitis-derived pseudoaneurysm six months after transplantation. A stent was placed in the iliac artery, resulting in a non-functioning pancreas, though the graft remained in situ. The other patient experienced partial pancreatic graft dysfunction, requiring two months of postoperative insulin therapy. Given the limited number of pancreas graft loss events and the heterogeneity of their underlying causes, these associations should be interpreted as descriptive and hypothesis-generating.

In addition to pancreas-related outcomes, kidney graft injury was also evaluated through the occurrence of ATN. In this context, elevated sVEGFR1 at 24 h PR and higher preoperative HS levels were associated with the development of kidney ATN. ATN occurred in four recipients during the early postoperative period. Importantly, three of these patients subsequently developed an episode of acute kidney rejection 19, 26 and 71 days post-transplantation, which represents a relatively late event compared with immediate post-transplant injury. Notably, endothelial biomarkers showed marked increases immediately after reperfusion or at 24 h, consistent with early endothelial activation due to IRI. Biomarkers had generally returned toward baseline values by the time of discharge, supporting resolution of acute endothelial response before any subsequent acute kidney rejection events. Therefore, ATN represented an early postoperative event, whereas acute rejection occurred later during the follow-up. The number of ATN cases is summarized in Table 4. Non-significant results are shown in Supplementary Tables S1–S3.

### 3.5. Endothelial Injury Markers Associated with Post-Transplant Complications in Functioning Grafts

In recipients with a functioning pancreas graft, higher hyaluronan levels at 24 h PR and discharge were associated with graft pancreatitis (Table 5). This condition was also associated with lower preoperative VEGF and sVEGFR1 levels. The occurrence of vascular thrombosis was associated with increased preoperative HS and reduced syndecan-1. Intestinal complications were linked to lower preoperative VEGF and HS levels at 24 h PR. Non-significant associations are provided in the Supplementary Tables S4–S9.

### 3.6. Postoperative Complications Associated with Pancreas Graft Loss

Abdominal collection, graft pancreatitis, vascular thrombosis, and kidney DGF were significantly associated with subsequent pancreas graft loss (Table 6).

Kidney surgical and urological complications were mainly represented by hematuria (*n* = 5) and perirenal hematoma (*n* = 5), whereas less frequent but more severe events included vascular thrombosis (*n* = 2) and urinary leakage (*n* = 2). Hematuria was typically mild to moderate and managed conservatively, except for one patient who required surgical intervention. Perirenal hematomas exhibited variable severity, being treated with interventional radiology in one case, surgery in another, and conservative measures in the remaining three. All cases of renal artery thrombosis resulted in graft nephrectomy. Urinary leaks were managed with ureterovesical reimplantation.

## 4. Discussion

To our knowledge, this is the first comprehensive study characterizing endothelial integrity and function in the context of IRI during pancreas transplantation. Previous experimental studies on pancreatic IRI have mainly focused on capillary density and leukocyte-endothelium interactions [24,25], whereas data on eGCX alterations in human pancreas transplantation remain lacking. Accordingly, our study was designed to capture both the immediate endothelial response after graft reperfusion and the dynamic evolution of endothelial injury throughout the perioperative period.

The observed increases in circulating endothelial injury markers were compatible with endothelial damage following transplantation. Syndecan-1, HS and sVEGFR1 rose significantly as early as 10 min PR, while VEGF concentrations sharply decreased at this same time point. This immediate post-reperfusion window predominantly reflects pancreas-specific injury, as the kidney graft had not yet been implanted. By contrast, biomarker changes observed at 24 h, including the elevation of hyaluronan, should be interpreted within the broader SPK context, as both grafts had been implanted by then and may contribute to the systemic endothelial response.

Ischemic injury induces VEGF release from endothelial cells while simultaneously increasing circulating sVEGFR1, a high-affinity VEGF inhibitor [26]. In our cohort, sVEGFR1 increased nearly 150-fold at 10 min PR relative to baseline, whereas VEGF decreased approximately 15-fold, suggesting rapid VEGF sequestration and reduced bioavailability during the immediate post-reperfusion phase. The limited availability at this stage may compromise endothelial repair and microvascular stability, thereby exacerbating IRI and compromising early graft perfusion.

In this predominantly SPK cohort, the elevated baseline sTM likely reflects pre-existing endothelial injury associated with diabetic kidney disease [27]. Supporting this interpretation, sTM correlated strongly with creatinine at 24 h PR (Supplementary Figure S1), with the highest concentrations in patients with longer pre-transplant dialysis duration. These findings are in keeping with the concept that endothelial dysfunction is already established before transplantation in many recipients with longstanding diabetes and renal impairment [28]. In line with this, patients in our cohort showed higher baseline syndecan-1, sTM, and VEGF compared with healthy controls, indicating underlying vascular injury prior to transplantation. In contrast, hyaluronan and sVEGFR1 were similar, whereas HS concentrations were lower. Previous reports have identified hyaluronan and syndecan-1 as reliable markers of eGCX shedding in diabetes [11]. Nonetheless, the extent of eGCX degradation likely varies among individuals, as common DM-related comorbidities such as dyslipidemia and hypertension also contribute to endothelial dysfunction [29].

When analyzed by donor type, cDCD grafts were associated with higher syndecan-1 at 10 min PR and increased VEGF at 24 h PR compared with DBD grafts, consistent with the more pronounced IRI associated with warm ischemia in cDCD donors [30,31,32]. Pancreas graft loss occurred in seven recipients, most within the first month after transplantation and primarily due to vascular thrombosis and graft pancreatitis. Lower plasma VEGF at 10 min PR and reduced hyaluronan at discharge were associated with graft loss, although independent associations could not be assessed due to the limited number of events.

Elevated sVEGFR1 has been linked to small-vessel dysfunction in kidney transplantation [33], and an imbalance in the VEGF-sVEGFR1 axis may promote endothelial injury and microvascular loss, contributing to ATN. In our cohort, increased sVEGFR1 plasma levels at 24 h PR were linked to ATN, suggesting systemic microvascular stress across the transplant setting. Some preoperative circulating eGCX markers were also associated with postoperative complications, including pancreas graft loss and kidney ATN, indicating that baseline endothelial profiling may have prognostic value in patients with diabetes. Notably, acute kidney rejection occurred after ATN onset, supporting the view that the early biomarker changes observed in this study primarily reflect IRI rather than subsequent immune-mediated events.

Kidney-related complications were also reflected by DGF, which was associated with poorer pancreas graft outcomes, highlighting the clinical interdependence of both grafts in SPK transplantation. Kidney postoperative morbidity in our cohort additionally included heterogeneous surgical/urological complications, most commonly hematuria and perirenal hematoma, whereas the two cases of renal artery thrombosis represented the most severe kidney surgical events and led to graft nephrectomy. Because of the low number of cases in each surgical subtype, these events were interpreted descriptively and were not analyzed separately in relation to circulating biomarkers.

Abdominal collections, graft pancreatitis and vascular thrombosis were also associated with pancreas graft loss, in agreement with the major postoperative complications known to compromise graft viability after transplantation [34].

Among recipients with functioning grafts, elevated hyaluronan at 24 h PR and lower preoperative VEGF and sVEGFR1 were linked to graft pancreatitis, a condition that may lead to pseudoaneurysm formation and life-threatening complications [35]. Vascular thrombosis was associated with lower baseline syndecan-1 and higher HS concentrations, consistent with observations reported in other pathological conditions [36,37]. These results further support the relevance of preoperative endothelial status in shaping postoperative outcomes.

The endothelial injury patterns identified in this study provide insight into possible pathways underlying graft injury and suggest potential avenues for graft protection. Glycocalyx degradation markers, together with alterations in the VEGF-sVEGFR1 axis, are consistent with early microvascular dysfunction during the perioperative period and support the relevance of these pathways as potential targets for strategies aimed at reducing IRI and preserving endothelial integrity. In parallel, optimization of graft preservation approaches, including ex situ machine perfusion [38,39], improved preservation solutions, and careful perioperative hemodynamic management, may help limit glycocalyx disruption and microvascular injury.

From a translational perspective, preoperative endothelial profiling may help identify recipients at increased risk of postoperative complications, while early post-reperfusion biomarker assessment may support more individualized perioperative management. These approaches warrant further investigation in pancreas transplantation, particularly in predominantly SPK cohorts.

Our study includes several aspects that warrant careful interpretation. First, the results are derived from a single-center cohort, which may affect their broader applicability. In addition, the relatively small sample size and low number of clinical events, particularly pancreatic graft loss, reduced statistical power and precluded multivariable analyses. Therefore, these findings should be regarded as primarily descriptive and hypothesis-generating, and external validation in larger, multicenter cohorts would be valuable. The study population did not encompass a kidney-only transplant cohort. Although such a comparator could provide useful contextual information regarding endothelial injury associated with renal ischemia–reperfusion, it would not allow a direct quantitative separation of pancreas- and kidney-derived endothelial injury in SPK, because renal reperfusion occurs after pancreas implantation within a distinct perioperative inflammatory and hemodynamic environment already shaped by pancreas reperfusion. Nonetheless, inclusion of a 10 min PR sampling time point before kidney implantation provides a unique opportunity to assess the immediate endothelial response predominantly attributable to pancreas reperfusion, thereby permitting partial distinction between early pancreas-specific injury and later systemic alterations in the SPK setting. In addition, the present study did not establish clinically applicable cut-off values or validated thresholds for the studied biomarkers, nor did it assess their predictive performance for post-transplant complications. Therefore, although these markers may be relevant for biological characterization and may inform future validation studies, their direct implementation in clinical decision-making is not yet supported.

A prospective design combined with structured perioperative sampling enabled characterization of circulating endothelial biomarker dynamics and suggests their potential utility as tools for perioperative monitoring and clinical risk assessment. These observations provide a rationale for further investigation of strategies aimed at preserving endothelial stability to improve pancreas graft outcomes and, particularly in predominantly SPK cohorts, to elucidate the interplay between pancreas and kidney graft injury in the early post-transplant period.

## 5. Conclusions

In summary, pancreas transplantation in a cohort largely composed of SPK recipients was associated with early endothelial injury and glycocalyx disruption. Perioperative changes in circulating endothelial biomarkers were linked to post-transplant morbidity and graft-specific outcomes. These findings support further evaluation of endothelial biomarkers for perioperative risk stratification in pancreas transplantation, although clinically applicable cut-off values remain to be established.

## Figures and Tables

**Table 1:** Demographic and clinicopathological features of donors.

	Donors (*N* = 52)
Age (years)	28 [19; 38.5]
Gender ratio (M/F)	38 (73.1)/14 (26.9)
Type of donor:	
-DBD	42 (80.8)
-cDCD	10 (19.2)
Cause of death:	
-Trauma	22 (42.3)
-Anoxic damage	10 (19.2)
-CVA	17 (32.7)
-Others	3 (5.8)
BMI (Kg/m^2^)	22 [21; 24.5]
Pancreas CIT (h)	7.7 [6.2; 9.9]
Kidney CIT (h)	10 [8.4; 12]
Preservation solution:	
-UW	3 (5.8)
-IGL-1	37 (71.2)
-CS	11 (21.1)
-HTK	1 (1.9)
ICU days	2 [1; 4.3]

Continuous variables are expressed as median [IQR] and categorical variables as frequencies (percentages). M indicates male; F, female; DBD, donor brain death; cDCD; controlled donor after cardiac death; CVA, cerebrovascular accident; BMI, body mass index; CIT, cold ischemia time; UW, University of Wisconsin; IGL-1, Institut Georges Lopez-1; CS, Celsior; HTK, Histidine-Tryptophan-Ketoglutarate; ICU, Intensive Care Unit.

**Table 2:** Demographic and clinicopathological features of recipients.

	Recipients (*N* = 52)
Age (years)	43 [36; 48]
Gender ratio (M/F)	29 (55.8)/23 (44.2)
BMI (kg/m^2^)	22.5 [21; 24.5]
DM vintage (years)	27 [22; 32]
Dialysis vintage (months)	6 [0; 16]
Dialysis type:	
-Hemodialysis	22 (42.3)
-Peritoneal Dialysis	12 (23.1)
-Pre-emptive	13 (25)
-No dialysis	5 (9.6)
Transplant type:	
-SPK	47 (90.4)
-PAK	-
-PA	-
-Pancreas retransplantation	5 (9.6)
Arterial anastomosis:	
-SA-SMA	36 (69.2)
-“Y” iliac graft; SA-SMA-iliac graft	16 (30.8)
Amylase (U/L)	175 [117; 275]
Lipase (U/L)	154 [101; 256]
Creatinine (mg/dL)	4.7 [2.7; 6.8]
LDH (U/L)	300 [232; 362]
Hospital stay (days)	11 [9; 15]

Continuous variables are expressed as median [IQR] and categorical variables as frequencies (percentages). M indicates male; F, female; BMI, body mass index; DM, Diabetes Mellitus; SPK, Simultaneous Pancreas-Kidney; PAK, Pancreas After Kidney; PA, Pancreas Transplant Alone; SA-SMA, Splenic Artery—Superior Mesenteric Artery; LDH, Lactate Dehydrogenase.

**Table 3:** Circulating markers of endothelial dysfunction in healthy controls and recipients at various transplant time points.

	Healthy Controls (*N* = 16)	Transplant Recipients (*N* = 52)				*p*
		BS	10 min PR	24 h PR	Discharge	
Syndecan-1 (ng/mL)	29.5 [24.7; 40]	48 [34; 83]	145 [83.7; 208.8]	44 [32; 69]	37 [27; 52]	<0.0001 0.0132 ^a^ <0.0001 ^b^ 0.0272 ^c^
Hyaluronan (ng/mL)	35.4 [18.6; 45.1]	23 [17; 35]	25 [18; 42]	46.5 [26.5; 87.2]	40 [27; 63]	<0.0001
HS (ng/mL)	6.3 [5.3; 7.1]	4.8 [3.8; 5.56]	6 [5.1; 7]	6.5 [5.3; 7.4]	6.4 [4.9; 7.6]	<0.0001 0.0031 ^a^
sTM (ng/mL)	3.2 [2.6; 3.5]	13.9 [10.8; 15.8]	12 [9.2; 15.2]	11.4 [9.2; 13.8]	8.1 [6.6; 9.1]	<0.0001 <0.0001 ^a^ <0.0001 ^b^ <0.0001 ^c^ <0.0001 ^d^
VEGF (pg/mL)	23 [17.7; 48.7]	74 [34; 114]	5 [0; 11]	31 [19; 49.2]	40 [29; 82]	<0.0001 0.0150 ^a^ 0.0011 ^b^
sVEGFR1 (pg/mL)	235.5 [193; 268]	202 [172; 264]	30,130 [21,928; 37,088]	438 [334; 649]	245 [210; 344]	<0.0001 <0.0001 ^b^ 0.0006 ^c^

Data are presented as median [IQR]. *p*-values of less than 0.05 were considered statistically significant. ^a^ Control vs. BS; ^b^ Control vs. 10 min PR; ^c^ Control vs. 24 h PR; ^d^ Control vs. Discharge. BS, before surgery; PR, post-reperfusion; HS; Heparan sulfate; sTM, soluble Thrombomodulin; VEGF, Vascular Endothelial Growth Factor; sVEGFR1, soluble Vascular Endothelial Growth Factor Receptor 1.

**Table 4:** Evolution of endothelial injury markers by donor type, pancreas graft loss, and kidney ATN development.

		**Donor type**		
	**Total (** ***N* = 52)**	**DBD (** ***n* = 42)**	**cDCD (** ***n* = 10)**	***p***
Syndecan-1 (10 min PR)	144.5 [84.5; 206.5]	117.5 [77; 190]	199.5 [174; 233]	0.0001
VEGF (24 h PR)	31 [19; 48.5]	25 [19; 44]	43 [33; 77]	0.038
		**Pancreas graft loss**		
	**Total (** ***N* = 52)**	**No (** ***n* = 45)**	**Yes (** ***n* = 7)**	***p***
Hyaluronan (BS)	23 [17; 35]	25 [19.5; 35.5]	17 [10; 25]	0.040
VEGF (10 min PR)	5 [0; 11]	5.5 [0; 11]	2 [0; 6]	0.040
Hyaluronan (Discharge)	40 [27; 62]	46 [27.5; 64]	30 [19; 35]	0.008
		**Kidney ATN**		
	**Total (** ***N* = 52)**	**No (** ***n* = 48)**	**Yes (** ***n* = 4)**	***p***
HS (BS)	4.8 [3.8; 5.6]	4.7 [3.8; 5.5]	5.5 [5.1; 5,9]	0.031
sVEGFR1 (24 h PR)	438 [334; 649]	437 [333; 634]	641 [493; 943]	0.020

Variables are expressed as median [IQR]. *p*-values of less than 0.05 were considered statistically significant. DBD indicates donation after brain death; cDCD; controlled donation after cardiac death; PR, post-reperfusion; HS, heparan sulfate; BS; before surgery; VEGF, vascular endothelial growth factor; sVEGFR1, soluble vascular endothelial growth factor receptor 1; ATN, acute tubular necrosis. Statistically non-significant results have not been included in this Table.

**Table 5:** Endothelial injury markers evolution in relation to post-transplant complications in recipients with functioning pancreas graft.

		**Graft pancreatitis**		
	**Total (** ***n* = 44)**	**No (*n* = 40)**	**Yes (*n* = 4)**	***p***
VEGF (BS)	76 [34; 108]	87 [40; 117]	33.5 [28.5; 55]	0.034
sVEGFR1 (BS)	202 [172; 269]	211 [174; 276]	180 [86; 198.5]	0.002
Hyaluronan (24 h PR)	38 [28; 88]	37 [26; 83]	137 [64; 188]	0.031
Hyaluronan (Discharge)	46 [27.5; 64]	45 [27; 62]	78 [36; 124]	0.005
		**Vascular thrombosis**		
	**Total (*n* = 44)**	**No (*n* = 34)**	**Yes (*n* = 10)**	***p***
HS (BS)	4.7 [3.8; 5.4]	4.7 [3.8; 5.3]	5.9 [4.1; 6.6]	0.029
Syndecan-1 (BS)	146 [86; 211]	147 [96; 233]	79.5 [64; 190]	0.039
		**Intestinal complications**		
	**Total (*n* = 44)**	**No (*n* = 40)**	**Yes (*n* = 4)**	***p***
VEGF (BS)	76 [34; 108]	87 [40; 117]	24 [18; 48]	0.005
HS (24 h PR)	6.4 [5.3; 7.4]	6.6 [5.3; 7.6]	5.7 [5.3; 6.1]	0.037

Variables are expressed as median [IQR]. *p*-values of less than 0.05 were considered statistically significant. BS indicates before surgery; PR, post-reperfusion; HS, heparan sulfate; VEGF, vascular endothelial growth factor; sVEGFR1, soluble vascular endothelial growth factor receptor 1. Statistically non-significant results have not been included in this Table.

**Table 6:** Postoperative complications and their association with pancreas graft loss.

		Pancreas Graft Loss *N* = 7		
	Total (*N* = 52)	No	Yes	*p*
Abdominal hemorrhage	9 (17.3)	6 (13.3)	3 (42.9)	0.090
Abdominal collection	8 (15.4)	4 (8.9)	4 (57.1)	**0.007**
Pancreas fistula	4 (7.7)	2 (4.4)	2 (28.6)	0.083
Graft pancreatitis	7 (13.5)	4 (8.9)	3 (42.9)	**0.043**
Vascular thrombosis	16 (30.8)	10 (22.2)	6 (85.7)	**0.002**
Intestinal complications	5 (15.4)	4 (8.9)	1 (14.3)	0.530
Kidney surgical/urological complications	14 (26.9)	13 (28.9)	1 (14.3)	0.659
Kidney DGF	9 (17.3)	5 (11.1)	4 (57.1)	**0.013**

Variables are expressed as frequencies (percentages). *p*-values < 0.05 in bold show significant differences between groups. DGF, delayed graft function.

## Data Availability

The original contributions presented in the study are included in the article/Supplementary Materials, further inquiries can be directed to the corresponding author.

## Supplementary Materials

The following supporting information can be downloaded at: https://www.mdpi.com/article/10.3390/medsci14020241/s1, Table S1: Evolution of endothelial injury markers by donor type; Table S2: Evolution of endothelial injury markers by pancreas graft loss development; Table S3: Evolution of endothelial injury markers by kidney ATN development; Table S4: Changes in endothelial injury markers according to the Clavien-Dindo classification in recipients with functioning pancreas grafts; Table S5: Changes in endothelial injury markers according to abdominal hemorrhage in recipients with functioning pancreas grafts; Table S6: Changes in endothelial injury markers according to graft pancreatitis in recipients with functioning pancreas grafts; Table S7: Changes in endothelial injury markers according to vascular thrombosis in recipients with functioning pancreas grafts; Table S8: Changes in endothelial injury markers according to intestinal complications in recipients with functioning pancreas grafts; Table S9: Changes in endothelial injury markers according to kidney delayed graft function in recipients with functioning pancreas grafts; Figure S1: Correlation of endothelial injury marker sTM and creatinine 24 h after grafts reperfusion.

## Abbreviations

The following abbreviations are used in this manuscript:

ATN	Acute Tubular Necrosis
DBD	Donor after Brain Death
cDCD	Controlled Donor after Circulatory Death
DGF	Delayed Graft Function
DM	Diabetes Mellitus
eGCX	endothelial Glycocalyx
HS	Heparan Sulfate
IRI	Ischemia–Reperfusion Injury
LDH	Lactate Dehydrogenase
PR	Post-Reperfusion
SPK	Simultaneous Pancreas-Kidney
sTM	soluble Thrombomodulin
VEGF	Vascular Endothelial Growth Factor
sVEGFR1	soluble Vascular Endothelial Growth Factor Receptor 1
