# Supplementary material for: Early Endothelial Injury in Pancreas Transplantation: Insights from a Prospective Cohort Largely Composed of Simultaneous Pancreas-Kidney Recipients
Source: Med Sci (Basel). 2026 May 6;14(2):241. doi: 10.3390/medsci14020241 (PMC13214961; doi:10.3390/medsci14020241)
Supplement: Supplementary file 1 [file medsci-14-00241-s001.zip › medsci-4286688-supplementary.pdf]

SUPPLEMENTARY MATERIAL

Supplementary Tables

Table S1. Evolution of endothelial injury markers by donor type. *page 2*

Table S2. Evolution of endothelial injury markers by pancreas graft loss development. *page 3*

Table S3. Evolution of endothelial injury markers by kidney ATN development. *page 4*

Table S4. Changes in endothelial injury markers according to the Clavien-Dindo classification in recipients with functioning pancreas grafts. *page 5*

Table S5. Changes in endothelial injury markers according to abdominal hemorrhage in recipients with functioning pancreas grafts. *page 6*

Table S6. Changes in endothelial injury markers according to graft pancreatitis in recipients with functioning pancreas grafts. *page 7*

Table S7. Changes in endothelial injury markers according to vascular thrombosis in recipients with functioning pancreas grafts. *page 8*

Table S8. Changes in endothelial injury markers according to intestinal complications in recipients with functioning pancreas grafts. *page 9*

Table S9. Changes in endothelial injury markers according to kidney delayed graft function in recipients with functioning pancreas grafts. *page. 10*

Supplementary Figure

Figure S1. Correlation of endothelial injury marker sTM and creatinine 24 h after grafts reperfusion. *page 11*

Table S1. Evolution of endothelial injury markers by donor type.

		DONOR TYPE		
	**Total (N=52)**	**DBD (n=42)**	**DCD (n=10)**	***p***
Syndecan-1 (24 h PR)	44 [32; 69]	44 [31; 63]	67.5 [39; 83]	0.196
Syndecan-1 (Discharge)	37 [27; 52]	36 [27; 46]	46.5 [27; 57]	0.422
VEGF (10 min PR)	5 [0; 11]	5 [0; 11]	5 [0; 8]	0.711
VEGF (Discharge)	40 [29; 82]	34 [29; 70]	53.5 [35; 12]	0.072
Hyaluronan (10 min PR)	25 [18; 42]	24 [18; 35]	30.5 [20; 4]	0.500
Hyaluronan (24 h PR)	46.5 [27; 86.5]	44.5 [26; 92]	54.5 [30; 83]	0.737
Hyaluronan (Discharge)	40 [27; 62]	45 [31; 62]	28.5 [25; 64]	0.272
HS (10 min PR)	6 [5.1; 7]	6.1 [5.1; 7]	5.6 [4.4; 6.4]	0.484
HS (24 h PR)	6.5 [5.3; 7.4]	6.5 [5.5; 7.8]	6 [5.1; 7.4]	0.432
HS (Discharge)	6.4 [4.9; 7.6]	6.2 [4.7; 7.]	6.8 [6.5; 8.9]	0.117
sTM (10 min PR)	12 [9.3; 15.2]	11.9 [8.4; 15.3]	12.5 [10.6; 13.3]	0.746
sTM 24 h PR)	11.4 [9.2; 13.7]	11.3 [9.1;14.2]	11.4 [9.9; 12.8]	0.996
sTM (Discharge)	8.1 [6.7; 9.1]	7.7 [6.7; 9]	8.4 [6.9; 9.1]	0.529
sVEGFR1 (10 min PR)	30 130 [21 928; 37 088]	31 436 [23 100; 39 222]	29 813 [21 780; 35 570]	0.430
sVEGFR1 (24 h PR)	438 [334; 649]	447 [334; 650]	414.5 [376; 514]	0.499
sVEGFR1 (Discharge)	245 [210; 344]	245 [210; 350]	262 [241; 309]	0.321

Variables are expressed as median [IQR]. DBD indicates donation after brain death; DCD; donation after cardiac death; PR, post-reperfusion; VEGF, vascular endothelial growth factor; HS, heparan sulfate; sTM, soluble thrombomodulin; sVEGFR1, soluble vascular endothelial growth factor receptor 1. Only statistically non-significant results (*p*-values ≥0.05) have been included in this table.

Table S2. Evolution of endothelial injury markers by pancreas graft loss.

		PANCREAS GRAFT LOSS		
	**Total (N=52)**	**No (n=45)**	**Yes (n=7)**	***p***
Syndecan-1 (BS)	48 [34; 83]	46 [34; 78]	58 [36; 126]	0.398
Syndecan-1 (10 min PR)	144.5 [84.5; 206.5]	146 [86; 211]	139 [52; 197]	0.630
Syndecan-1 (24 h PR)	44 [32; 69]	44 [31; 66]	49 [39; 93]	0.481
Syndecan-1 (Discharge)	37 [27; 52]	37 [26; 48]	44 [31; 62]	0.479
VEGF (BS)	74 [34; 112]	76 [34; 108]	66 [51; 116]	0.594
VEGF (24 h PR)	31 [19; 48]	30 [19; 46]	33 [25; 43]	0.549
VEGF (Discharge)	40 [29; 82]	35 [29; 70]	99 [35; 151]	0.217
Hyaluronan (10 min PR)	25 [18; 42]	24 [17; 36]	28 [22; 86]	0.214
Hyaluronan (24 h PR)	46.5 [27; 86.5]	38 [28; 88]	67 [24; 85]	0.560
HS (BS)	4.8 [3.8; 5.6]	4.7 [3.8; 5.4]	4.9 [3.8; 6.1]	0.518
HS (10 min PR)	6 [5.1; 7]	6.1 [5.1; 7]	5.5 [5.1; 6.9]	0.941
HS (24 h PR)	6.5 [5.3; 7.4]	6.4 [5.25; 7.4]	6.5 [5.5; 7.5]	0.661
HS (Discharge)	6.4 [4.9; 7.6]	6.4 [5.1; 7.9]	6.3 [3.9; 6.5]	0.366
sTM (BS)	13.9 [10.8; 15.7]	13.9 [10.8; 15.9]	12 [9.5; 15.2]	0.723
sTM (10 min PR)	12 [9.3; 15.2]	12.5 [9.5; 15.3]	10.6 [8.3; 13.3]	0.409
sTM 24 h PR)	11.4 [9.2; 13.8]	11.3 [9.2; 13.7]	11.4 [9.6; 15]	0.818
sTM (Discharge)	8.1 [6.7; 9.1]	7.7 [6.7; 9]	8.6 [7.3; 11.9]	0.774
sVEGFR1 (BS)	202 [172; 264]	202 [172; 269]	204.5 [194; 211]	0.143
sVEGFR1 (10 min PR)	30 130 [29 928; 37 088]	30 783 [21 854; 38 155]	28 164 [24 420; 35 572]	0.634
sVEGFR1 (24 h PR)	438 [334; 649]	437.5 [336.5; 649.5]	565 [290; 620]	0.733
sVEGFR1 (Discharge)	245 [210; 344]	262 [214; 343]	242 [210; 545]	0.842

Variables are expressed as median [IQR]. BS, before surgery; PR, post-reperfusion; VEGF, vascular endothelial growth factor; HS, heparan sulfate; sTM, soluble thrombomodulin; sVEGFR1, soluble vascular endothelial growth factor receptor 1. Only statistically non-significant results (*p*-values ≥0.05) have been included in this table.

Table S3. Evolution of endothelial injury markers by kidney ATN development.

		KIDNEY ATN		
	**Total (N=52)**	**No (n=48)**	**Yes (n=4)**	***p***
Syndecan-1 (BS)	48 [34; 83]	52 [34; 83]	30 [19.5; 73.5]	0.266
Syndecan-1 (10 min PR)	144.5 [84.5; 206.5]	146.5 [91; 206.5]	59 [49; 150]	0.076
Syndecan-1 (24 h PR)	44 [32; 69]	47 [32; 69]	34 [25.5; 59.5]	0.243
Syndecan-1 (Discharge)	37 [27; 52]	38 [26; 57]	36.5 [34.5; 37.5]	0.184
VEGF (BS)	74 [34; 112]	74 [34; 116.5]	73 [37.5; 96]	0.822
VEGF (10 min PR)	5 [0; 11]	5 [0; 11]	4.5 [2; 7.5]	0.158
VEGF (24 h PR)	31 [19; 48.5]	31 [19; 48.5]	29 [17.5; 72]	0.753
VEGF (Discharge)	40 [29; 82]	40 [30; 82]	37 [27; 144.5]	0.930
Hyaluronan (BS)	23 [17; 35]	22 [17; 35]	25.5 [24; 34.5]	0.428
Hyaluronan (10 min PR)	25 [18; 42]	26 [18; 42]	19.5 [18; 31]	0.237
Hyaluronan (24 h PR)	46.5 [27; 86.5]	44.5 [27; 90]	54.5 [33.5; 68]	0.850
Hyaluronan (Discharge)	40 [27; 62]	42 [27; 62]	31 [18; 78]	0.692
HS (10 min PR)	6 [5.1; 7]	6 [5.1; 6.9]	6.3 [5.3; 7.1]	0.598
HS (24 h PR)	6.5 [5.3; 7.4]	6.5 [5.2; 7.5]	5.9 [5.7; 6.6]	0.652
HS (Discharge)	6.4 [4.9; 7.6]	6.4 [4.7; 7.6]	6.7 [5.8; 8.9]	0.322
sTM (BS)	13.9 [10.8; 15.7]	13.9 [10.8; 15.3]	14.2 [9; 16.7]	0.941
sTM (10 min PR)	12 [9.3; 15.2]	12 [9.3; 15.2]	12.2 [9.2; 14.4]	0.980
sTM (24 h PR)	11.4 [9.2; 13.7]	11.4 [9.2;13.5]	12.5 [10; 14.2]	0.511
sTM (Discharge)	8.1 [6.7; 9.1]	7.7 [6.5; 9]	9.9 [8; 11.3]	0.109
sVEGFR1 (BS)	202 [172; 264]	209 [166; 264]	200.5 [196.5; 264.5]	0.975
sVEGFR1 (10 min PR)	30 130 [21 928; 37 088]	30 130 [21 780; 36 810]	32 994 [28 532; 52 004]	0.198
sVEGFR1 (Discharge)	245 [210; 344]	244 [203; 344]	285 [241.5; 427]	0.452

Variables are expressed as median [IQR]. BS, before surgery; PR, post-reperfusion; VEGF, vascular endothelial growth factor; HS, heparan sulfate; sTM, soluble thrombomodulin; sVEGFR1, soluble vascular endothelial growth factor receptor 1. Only statistically non-significant results (p-values ≥0.05) have been included in this table.

Table S4. Changes in endothelial injury markers according to the Clavien-Dindo classification in recipients with functioning pancreas grafts.

		**CLAVIEN-DINDO**		
	**Total (n=44)**	**<IIIa (n=39)**	**≥IIIa (n=5)**	***p***
Syndecan-1 (BS)	46 [33.5; 78]	45 [34; 80]	57 [30; 76]	0.998
Syndecan-1 (10 min PR)	146 [86; 211]	144.5 [84; 197]	234 [118; 288]	0.076
Syndecan-1 (24 h PR)	44 [31.5; 66.5]	44 [32; 67]	47 [27; 55]	0.581
Syndecan-1 (Discharge)	37 [26; 48]	37.5 [26.5; 47]	34 [21; 59]	0.905
VEGF (BS)	76 [34; 108]	86.5 [37; 124]	33 [24; 72]	0.114
VEGF (10 min PR)	5.5 [0; 11]	5 [0; 12]	6 [5; 10]	0.624
VEGF (24 h PR)	30 [19; 46]	28.5 [18.5; 48.5]	35 [23; 36]	0.953
VEGF (Discharge)	35 [29; 70]	34 [29; 70]	81 [41; 148]	0.131
Hyaluronan (BS)	25 [19.5; 35.5]	25 [20; 36]	22 [15; 26]	0.698
Hyaluronan (10 min PR)	24 [17; 36]	25 [18; 37]	20 [14; 22]	0.544
Hyaluronan (24 h PR)	38 [28; 88]	36.5 [27; 85.5]	72 [42; 97]	0.547
Hyaluronan (Discharge)	46 [27; 64]	47.5 [29.5; 64]	29 [17.5; 82]	0.487
HS (BS)	4.7 [3.8; 5.4]	4.7 [3.8; 5.5]	5.3 [3.9; 5.3]	0.597
HS (10 min PR)	6.1 [5.1; 7]	5.9 [4.9; 6.9]	6.5 [6.1; 7.1]	0.075
HS (24 h PR)	6.4 [5.3; 7.4]	6.5 [5.2; 7.4]	5.9 [5.5; 7.4]	0.953
HS (Discharge)	6.4 [5.1; 7.9]	6.4 [5.2; 7.9]	6.5 [4.1; 7.1]	0.714
sTM (BS)	13.9 [10.8; 15.9]	13.9 [10.8; 15.8]	14.2 [13.2; 15.9]	0.953
sTM (10 min PR)	12.5 [9.6; 15.3]	12.5 [9.3; 15.3]	12.5 [11.6; 13.1]	0.795
sTM (24 h PR)	11.3 [9.2; 13.7]	11.2 [9.2; 13.5]	12.1 [11.3; 14.2]	0.661
sTM (Discharge)	7.7 [6.7; 9]	7.7 [6.7; 9]	7.7 [6; 9.6]	0.523
sVEGFR1 (BS)	202 [172; 269]	210 [174.5; 272.5]	164 [158; 188]	0.153
sVEGFR1 (10 min PR)	30 783 [21 854; 38 155]	30 130 [21 780; 39 222]	35 570 [25 764; 37 088]	0.596
sVEGFR1 (24 h PR)	437.5 [336.5; 649.5]	438 [339; 649]	389 [305; 813]	0.895
sVEGFR1 (Discharge)	262 [214; 343]	253.5 [215.5; 342]	309 [201; 413]	0.781

Variables are expressed as median [IQR]. BS, before surgery; PR, post-reperfusion; VEGF, vascular endothelial growth factor; HS, heparan sulfate; sTM, soluble thrombomodulin; sVEGFR1, soluble vascular endothelial growth factor receptor 1. Only statistically non-significant results (p-values ≥0.05) have been included in this table.

Table S5. Changes in endothelial injury markers according to abdominal hemorrhage in recipients with functioning pancreas grafts.

		**ABDOMINAL HEMORRHAGE**		
	**Total (n=44)**	**No (n=38)**	**Yes (n=6)**	***p***
Syndecan-1 (BS)	46 [33.5; 78]	46 [34; 80]	45 [32; 76]	0.750
Syndecan-1 (10 min PR)	146 [86; 211]	146 [86; 202]	176 [64; 288]	0.823
Syndecan-1 (24 h PR)	44 [31.5; 66.5]	44 [31.5; 66]	51 [43; 82]	0.447
Syndecan-1 (Discharge)	37 [26; 48]	37 [26; 46]	46.5 [26; 60]	0.593
VEGF (BS)	76 [34; 108]	86 [40; 117]	31 [24; 93]	0.121
VEGF (10 min PR)	5.5 [0; 11]	5 [0; 12]	6 [4; 10]	0.331
VEGF (24 h PR)	30 [19; 46]	32 [19; 50]	23.5 [15; 35]	0.159
VEGF (Discharge)	35 [29; 70]	34.5 [29.5; 70]	54 [28; 82]	0.753
Hyaluronan (BS)	25 [19.5; 35.5]	25.5 [20; 36]	23 [15; 26]	0.613
Hyaluronan (10 min PR)	24 [17; 36]	24.5 [18; 35]	22 [14; 37]	0.850
Hyaluronan (24 h PR)	38 [28; 88]	37 [26; 83]	69.5 [28; 125]	0.656
Hyaluronan (Discharge)	46 [27.5; 64]	47 [31; 64]	18 [18; 55]	0.254
HS (BS)	4.7 [3.8; 5.4]	4.7 [3.8; 5.4]	5.3 [3.8; 6.6]	0.256
HS (10 min PR)	6.1 [5.1; 7]	5.9 [4.7; 7]	6.1 [5.9; 7]	0.192
HS (24 h PR)	6.4 [5.3; 7.4]	6.3 [5.2; 7.4]	6.7 [5.5; 7.4]	0.865
HS (Discharge)	6.4 [5.1; 7.9]	5.4 [5.2; 7.8]	61 [4.1; 9.3]	0.768
sTM (BS)	13.9 [10.8; 15.9]	13.8 [10.8; 16.2]	14.1 [9.4; 15.9]	0.718
sTM (10 min PR)	12.5 [9.6; 15.3]	12.9 [9.8; 15.3]	10.8 [8.3; 13.1]	0.297
sTM (24 h PR)	11.3 [9.2; 13.7]	11.3 [9.2; 13.7]	11.3 [9.7; 14.2]	0.895
sTM (Discharge)	7.7 [6.7; 9]	7.6 [6.7; 8.9]	9.1 [5.7; 9.8]	0.396
sVEGFR1 (BS)	202 [172; 269]	202 [172; 276]	202 [164; 262]	0.518
sVEGFR1 (10 min PR)	30 783 [21 854; 38 155]	30 783 [21 780; 39 222]	30 824 [25 764; 37 088]	0.802
sVEGFR1 (24 h PR)	437.5 [336.5; 649.5]	430.5 [334; 634]	636.5 [389; 813]	0.216
sVEGFR1 (Discharge)	262 [214; 343]	245 [214: 341]	361 [195; 581]	0.450

Variables are expressed as median [IQR]. BS, before surgery; PR, post-reperfusion; VEGF, vascular endothelial growth factor; HS, heparan sulfate; sTM, soluble thrombomodulin; sVEGFR1, soluble vascular endothelial growth factor receptor 1 Only statistically non-significant results (*p*-values ≥0.05) have been included in this table.

Table S6. Changes in endothelial injury markers according to graft pancreatitis in recipients with functioning pancreas grafts.

		**GRAFT PANCREATITIS**		
	**Total (n=44)**	**No (n=40)**	**Yes (n=4)**	***p***
Syndecan-1 (BS)	46 [33.5; 78]	45 [33.5; 81.5]	55 [33; 66.5]	0.884
Syndecan-1 (10 min PR)	146 [86; 211]	146 [86; 202]	171.5 [80; 298.5]	0.895
Syndecan-1 (24 h PR)	44 [31.5; 66.5]	44 [31.5; 68]	45.5 [34; 61]	0.406
Syndecan-1 (Discharge)	37 [26; 48]	37 [27; 46]	39 [19; 61]	0.993
VEGF (10 min PR)	76 [34; 108]	87 [40; 117]	33.5 [28.5; 55]	0.137
VEGF (24 h PR)	30 [19; 46]	30 [19; 47]	31 [19.5; 35.5]	0.663
VEGF (Discharge)	35 [29; 70]	34 [29; 70]	48 [36; 121]	0.329
Hyaluronan (BS)	25 [19.5; 35.5]	24 [19.5; 34.5]	38 [25.5; 95.5]	0.235
Hyaluronan (10 min PR)	24 [17; 36]	24 [18.5; 36]	19.5 [11.5; 79.5]	0.848
HS (BS)	4.7 [3.8; 5.4]	4.7 [3.8; 5.5]	4.6 [4; 5.2]	0.420
HS (10 min PR)	6.1 [5.1; 7]	6.2 [5.1; 7.1]	5.7 [4.7; 6]	0.151
HS (24 h PR)	6.4 [5.3; 7.4]	6.4 [5.4; 7.4]	6.3 [5.1; 7.8]	0.972
HS (Discharge)	6.4 [5.1; 7.9]	6.4 [5.1; 7.6]	6.7 [4.1; 9.4]	0.965
sTM (BS)	13.9 [10.8; 15.9]	13.9 [11.3; 16.2]	12.4 [8.7; 15]	0.481
sTM (10 min PR)	12.5 [9.6; 15.3]	12.9 [9.8; 15.3]	9.4 [6.3; 14.2]	0.352
sTM (24 h PR)	11.3 [9.2; 13.7]	11.3 [9.2; 13.7]	10.3 [8.1; 15.3]	0.989
sTM (Discharge)	7.7 [6.7; 9]	7.9 [6.7; 9]	7.4 [6.5; 10.1]	0.311
sVEGFR1 (10 min PR)	30 783 [21 854; 38 155]	30 783 [22 514; 38 155]	28 613 [16 430; 39 195]	0.756
sVEGFR1 (24 h PR)	437.5 [336.5; 649.5]	442 [336.5; 649.5]	372 [320; 601]	0.509
sVEGFR1 (Discharge)	262 [214; 343]	283 [217: 343]	187.5 [147; 313.5]	0.163

Variables are expressed as median [IQR]. BS, before surgery; PR, post-reperfusion; VEGF, vascular endothelial growth factor; HS, heparan sulfate; sTM, soluble thrombomodulin; sVEGFR1, soluble vascular endothelial growth factor receptor 1. Only statistically non-significant results (*p*-values ≥0.05) have been included in this table.

Table S7. Changes in endothelial injury markers according to vascular thrombosis in recipients with functioning pancreas grafts.

		**VASCULAR THROMBOSIS**		
	**Total (n=44)**	**No (n=34)**	**Yes (n=10)**	***p***
Syndecan-1 (10 min PR)	146 [86; 211]	147 [96; 233]	79.5 [64; 190]	0.089
Syndecan-1 (24 h PR)	44 [31.5; 66.5]	45.5 [32; 67]	40.5 [27; 63]	0.759
Syndecan-1 (Discharge)	37 [26; 48]	36.5 [26; 47]	39 [27; 57]	0.622
VEGF (BS)	76 [34; 108]	71 [32; 108]	96 [40; 131]	0.227
VEGF (10 min PR)	5.5 [0; 11]	6 [1; 12]	3 [0; 11]	0.935
VEGF (24 h PR)	30 [19; 46]	35 [19; 50]	22 [18; 36]	0.182
VEGF (Discharge)	35 [29; 70]	37.5 [28.5; 70.5]	34 [30; 70]	0.991
Hyaluronan (BS)	25 [19.5; 35.5]	23.5 [20; 34]	30 [12; 43]	0.734
Hyaluronan (10 min PR)	24 [17; 36]	24 [18; 37]	245 [9; 30]	0.528
Hyaluronan (24 h PR)	38 [28; 88]	42 [28; 97]	35 [25; 83]	0.657
Hyaluronan (Discharge)	46 [27.5; 64]	47.5 [27; 64]	34.5 [28; 61]	0.593
HS (10 min PR)	6.1 [5.1; 7]	6.1 [5.3; 6.8]	5.6 [4.7; 7.5]	0.899
HS (24 h PR)	6.4 [5.3; 7.4]	6.5 [5.2; 7.4]	6.1 [5.6; 7.3]	0.940
HS (Discharge)	6.4 [5.1; 7.9]	6.55 [5.1; 7.9]	5.6 [5.1; 7.6]	0.525
sTM (BS)	13.9 [10.8; 15.9]	14.1 [11.8; 16.2]	11.2 [8; 14.6]	0.152
sTM (10 min PR)	12.5 [9.6; 15.3]	13 [10.4; 15.3]	9.7 [6.3; 13.6]	0.088
sTM (24 h PR)	11.3 [9.2; 13.7]	11.8 [9.2; 13.8]	10.7 [7.7; 13.3]	0.331
sTM (Discharge)	7.7 [6.7; 9]	7.7 [6.5; 9.1]	7.8 [6.9; 8.9]	0.508
sVEGFR1 (BS)	202 [172; 269]	198 [147; 269]	209 [181; 346]	0.343
sVEGFR1 (10 min PR)	30 783 [21 854; 38 155]	32 610 [21 928; 39 222]	26 022 [15 204; 28 900]	0.292
sVEGFR1 (24 h PR)	437.5 [336.5; 649.5]	437 [334; 634]	433.5 [413; 709]	0.572
sVEGFR1 (Discharge)	262 [214; 343]	264 [203: 337.5]	262 [221; 479]	0.272

Variables are expressed as median [IQR]. BS, before surgery; PR, post-reperfusion; VEGF, vascular endothelial growth factor; HS, heparan sulfate; sTM, soluble thrombomodulin; sVEGFR1, soluble vascular endothelial growth factor receptor 1. Only statistically non-significant results (*p*-values ≥0.05) have been included in this table.

Table S8. Changes in endothelial injury markers according to intestinal complications in recipients with functioning pancreas grafts.

		INTESTINAL COMPLICATIONS		
	**Total (n=44)**	**No (n=40)**	**Yes (n=4)**	***p***
Syndecan-1 (BS)	46 [33.5; 78]	46 [34; 77]	53 [28.5; 93]	0.979
Syndecan-1 (10 min PR)	146 [86; 211]	146 [86; 202]	163 [88; 303]	0.476
Syndecan-1 (24 h PR)	44 [31.5; 66.5]	44 [31.5; 66.5]	43.5 [28; 68.5]	0.649
Syndecan-1 (Discharge)	37 [26; 48]	37 [27; 48]	38.5 [26; 54]	1.000
VEGF (10 min PR)	5.5 [0; 11]	5.5 [0; 11]	7.5 [4.5; 11.5]	0.966
VEGF (24 h PR)	30 [19; 46]	30 [19; 47]	25 [10.5; 39.5]	0.418
VEGF (Discharge)	35 [29; 70]	35 [30; 70]	68 [19; 148]	0.878
Hyaluronan (BS)	25 [19.5; 35.5]	25 [19.5; 35.5]	24 [18; 88.5]	0.857
Hyaluronan (10 min PR)	24 [17; 36]	24 [16; 35]	52 [21; 108]	0.254
Hyaluronan (24 h PR)	38 [28; 88]	37 [26; 88]	57 [37; 124.5]	0.189
Hyaluronan (Discharge)	46 [27.5; 64]	48 [28; 64]	40 [18; 44]	0.559
HS (BS)	4.7 [3.8; 5.4]	4.8 [3.8; 5.5]	4.3 [3.9; 5]	0.197
HS (10 min PR)	6.1 [5.1; 7]	5.9 [5; 7]	6.3 [5.8; 6.8]	0.266
HS (Discharge)	6.4 [5.1; 7.9]	6.4 [5.1; 7.9]	6.5 [4.7; 6.8]	0.731
sTM (BS)	13.9 [10.8; 15.9]	13.9 [10.8; 15.9]	13.7 [9; 15.2]	0.690
sTM (10 min PR)	12.5 [9.6; 15.3]	12.8 [9.6; 15.3]	12 [8.3; 12,8]	0.369
sTM (24 h PR)	11.3 [9.2; 13.7]	11.2 [9.2; 13.4]	12.8 [8.9; 16.3]	0.654
sTM (Discharge)	7.7 [6.7; 9]	7.9 [6.7; 9]	7.4 [6.3; 9.1]	0.510
sVEGFR1 (BS)	202 [172; 269]	202 [174; 264]	242.5 [79; 1532]	0.586
sVEGFR1 (10 min PR)	30 783 [21 854; 38 155]	30 125 [21 854; 36 190]	39 954 [29 067; 57 636]	0.196
sVEGFR1 (24 h PR)	437.5 [336.5; 649.5]	430.5 [336.5; 628.5]	995 [556; 1898.5]	0.206
sVEGFR1 (Discharge)	262 [214; 343]	245 [214: 341]	361 [255; 833]	0.205

Variables are expressed as median [IQR]. BS, before surgery; PR, post-reperfusion; VEGF, vascular endothelial growth factor; HS, heparan sulfate; sTM, soluble thrombomodulin; sVEGFR1, soluble vascular endothelial growth factor receptor 1. Only statistically non-significant results (*p*-values ≥0.05) have been included in this table.

Table S9. Changes in endothelial injury markers according to kidney delayed graft function in recipients with functioning pancreas grafts.

		**KIDNEY DELAYED GRAFT FUNCTION**		
	**Total (n=44)**	**No (n=39)**	**Yes (n=5)**	***p***
Syndecan-1 (BS)	46 [33.5; 78]	46 [34; 80]	45 [32; 76]	0.326
Syndecan-1 (10 min PR)	146 [86; 211]	146 [86; 202]	176 [64; 288]	0.456
Syndecan-1 (24 h PR)	44 [31.5; 66.5]	44 [31.5; 66]	51 [43; 82]	0.142
Syndecan-1 (Discharge)	37 [26; 48]	37 [26; 46]	46.5 [26; 60]	0.196
VEGF (BS)	76 [34; 108]	86 [40; 117]	31 [24; 93]	0.717
VEGF (10 min PR)	5.5 [0; 11]	5 [0; 12]	6 [4; 10]	0.499
VEGF (24 h PR)	30 [19; 46]	32 [19; 50]	23.5 [15; 35]	0.494
VEGF (Discharge)	35 [29; 70]	34.5 [29.5; 70]	54 [28; 82]	0.483
Hyaluronan (BS)	25 [19.5; 35.5]	25.5 [20; 36]	23 [15; 26]	0.655
Hyaluronan (10 min PR)	24 [17; 36]	24.5 [18; 35]	22 [14; 37]	0.836
Hyaluronan (24 h PR)	38 [28; 88]	37 [26; 83]	69.5 [28; 125]	0.951
Hyaluronan (Discharge)	46 [27.5; 64]	47 [31; 64]	18 [18; 55]	0.315
HS (BS)	4.7 [3.8; 5.4]	4.7 [3.8; 5.4]	5.3 [3.8; 6.6]	0.171
HS (10 min PR)	6.1 [5.1; 7]	6 [4.9; 6.9]	6.5 [5.7; 7.1]	0.319
HS (24 h PR)	6.4 [5.3; 7.4]	6.3 [5.2; 7.4]	6.7 [5.5; 7.4]	0.763
HS (Discharge)	6.4 [5.1; 7.9]	5.4 [5.2; 7.8]	61 [4.1; 9.3]	0.088
sTM (BS)	13.9 [10.8; 15.9]	13.8 [10.8; 16.2]	14.1 [9.4; 15.9]	0.804
sTM (10 min PR)	12.5 [9.6; 15.3]	12.9 [9.8; 15.3]	10.8 [8.3; 13.1]	0.925
sTM (24 h PR)	11.3 [9.2; 13.7]	11.3 [9.2; 13.7]	11.3 [9.7; 14.2]	0.641
sTM (Discharge)	7.7 [6.7; 9]	7.6 [6.7; 8.9]	9.1 [5.7; 9.8]	0.818
sVEGFR1 (BS)	202 [172; 269]	202 [172; 276]	202 [164; 262]	0.308
sVEGFR1 (10 min PR)	30 783 [21 854; 38 155]	30 783 [21 780; 39 222]	30 824 [25 764; 37 088]	0.638
sVEGFR1 (24 h PR)	437.5 [336.5; 649.5]	438 [334; 649]	421 [413; 709]	0.665
sVEGFR1 (Discharge)	262 [214; 343]	245 [214: 341]	361 [195; 581]	0.737

Variables are expressed as median [IQR]. BS, before surgery; PR, post-reperfusion; VEGF, vascular endothelial growth factor; HS, heparan sulfate; sTM, soluble thrombomodulin; sVEGFR1, soluble vascular endothelial growth factor receptor 1. Only statistically non-significant results (*p*-values ≥0.05) have been included in this table.

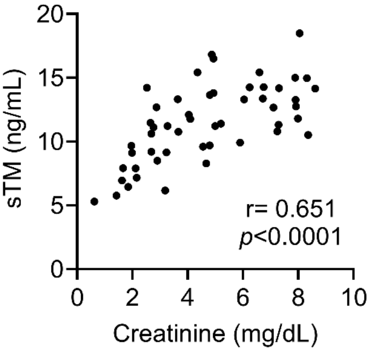

Figure S1. Correlation of endothelial injury marker sTM and creatinine 24 h after grafts reperfusion. Spearman's rank correlation coefficient (r) and p-value is indicated. *p*-values of less than 0.05 were statistically significant. sTM, soluble thrombomodulin.
